# Coupling of Intracellular Calcium Homeostasis and Formation and Secretion of Matrix Vesicles: Their Role in the Mechanism of Biomineralization

**DOI:** 10.3390/cells14100733

**Published:** 2025-05-17

**Authors:** Azzurra Margiotta

**Affiliations:** Department of Clinical Dentistry, Faculty of Medicine, University of Bergen, 5009 Bergen, Norway; azzurra198@libero.it

**Keywords:** osteoblastogenesis, osteogenesis, calcium signaling, matrix vesicles, mineralization

## Abstract

The human bone is a dynamic, highly vascularized tissue composed of 60–70% minerals, which include mainly calcium phosphate (CaP) in the form of hydroxyapatite (HA) crystals, 30% organic matrix composed of type I collagen fibers, and less than 5% water and lipids. The crystals are formed inside the matrix vesicles (MVs) and are then released in the organic collagen-based fibrous matrix. Extracellular matrix (ECM) formation and mineralization processes, named osteogenesis, are associated with human mesenchymal stem cells (hMSCs) undergoing differentiation into osteoblasts (osteoblastogenesis). Osteogenesis is regulated by multiple intracellular signaling and genetic pathways and by environmental factors. Calcium flow is finely regulated and plays a key role in both osteoblastogenesis and osteogenesis. The formation and accumulation of CaP, the biogenesis of MVs, their secretion, and the deposition of HA crystals to fill the organic bone matrix are the fundamental events in the biomineralization process. In this paper, I will describe and discuss the recent findings and hypothesis on the molecular mechanism regulating this process.

## 1. Cellular, Molecular, and Genetic Aspects of Bone Formation

Human bone tissue is made of various cells, has its own blood vessels, and is able to remodel itself in order to define the mass, morphology, and metabolic characteristics of the skeleton [1].

Osteoblasts, osteocytes, and osteoclasts are the three cell types that are fundamental for the development, growth, repair, and remodeling of bones, and they contribute to bone homeostasis. Osteoblasts are defined as bone-forming cells and are responsible for bone matrix formation and mineralization, osteocytes are mature bone cells, while the cells that break down bone are named osteoclasts [2].

Bone formation is a multi-step process, which occurs through the differentiation of mesenchymal stem cells (MSCs) into osteoblasts (osteoblastogenesis), their modulation by other cell types, including immune cells, the production of extracellular matrix (ECM), the biogenesis and release of matrix vesicles (MVs) and ECM mineralization [3].

**Figure 1:**
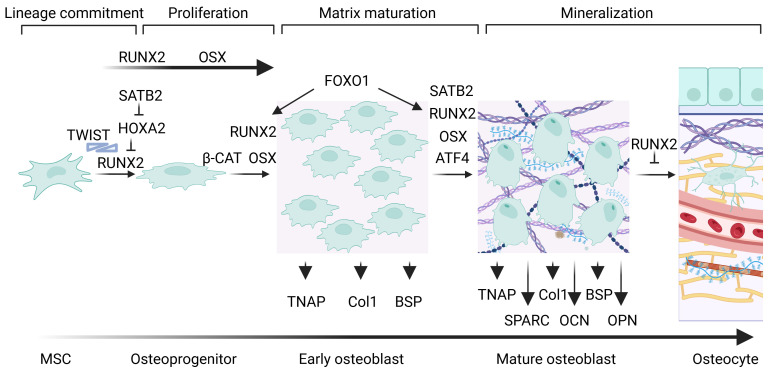
Transcriptional regulation during osteoblastogenesis and osteogenesis. Runx2 is the first transcription factor required for the differentiation of MSCs into osteoblasts. It induces the commitment of the osteoblast lineage, the differentiation of MSCs into early osteoblasts promoting osteoid formation, and it triggers the expression of osteogenic genes. Osx further regulate at later steps the differentiation of MSCs into mature osteoblasts. The *TNAP*, *Col1*, and *BSP* osteogenic genes are upregulated in early osteoblasts, while *SPARC* (coding for osteonectin), *OCN* (coding for osteocalcin) and *OPN* are upregulated in mature osteoblasts.

Osteoblastogenesis is a growth and differentiation program which is divided into four stages characterized by different markers: lineage commitment into osteoprogenitors, proliferation of osteoblast precursors, differentiation in early osteoblasts, which are responsible for extracellular matrix maturation, and differentiation in mature osteoblasts, which are linked to matrix mineralization [4,5]. Osteoblast differentiation is regulated by three transcription factors, Runt-related transcription factor 2 (also known as Runx2, coded by *RUNX2* gene), osterix (Osx, coded by *SP7/OSX* gene) and ATF-4 (cyclic AMP-dependent transcription factor, coded by *ATF4* gene), and is controlled at multiple levels such as transcriptional co-factors, inhibitors, osteo-enhancing and -suppressing miRNAs, epigenetics, systemic factors, hormonal controls, and environmental cues, such as light–dark cycle (Figure 1) [6].

### 1.1. The “Master Regulator of Osteogenesis”: Runx2

Runx2 is fundamental for osteoblast differentiation and chondrocyte maturation. In relation to osteoblastogenesis, *RUNX2* is expressed in uncommitted MSCs, upregulated in osteoblast precursors, highly upregulated in immature osteoblasts, and downregulated in mature osteoblasts [6,7]. The *RUNX2* gene expression is regulated by transcriptional factors, among which MSX-2, DLX-3, DLX-5, SATB2 (Special AT-rich sequence binding protein 2), FoxO1 (Forkhead box protein O1), Bapx1, PPARγ2 (regulator of adipocyte differentiation peroxisome proliferator-activated receptor γ 2) and Hox-A2 [6,8]. SATB2 mediates osteoblast proliferation in the early steps of osteoblastogenesis and contributes to the regulation of differentiation at later stages [6].

Moreover, Indian hedgehog (Ihh)/Gli2 signaling favors MSCs differentiation in osteoblast precursor by regulating the expression and stimulating Runx2 osteoblastogenic function [7]. Furthermore, Runx2 is subjected to the control of Wnt and transforming growth factor-β (TGF-β)/bone morphogenic protein (BMP) signaling pathways [9,10]. Interestingly, Wnt signaling activates the upregulation of *RUNX2*, *OSX*, and *DLX5* through the action of β-catenin in the canonical pathway and nuclear factor of activated T cells (NFAT) and phospho-c-JUN in the non-canonical pathway [9]. Similarly, both TGF-β and BMP signaling activate the canonical Smad-dependent pathway, with the phosphorylation of specific Smad proteins, which translocate into the nucleus to promote transcription, and the non-canonical Smad-independent p38 MAPK (mitogen-activated protein kinase) signaling pathway. Both pathways converge at the *RUNX2* gene in order to trigger the expression of osteoblast specific genes [9,10].

Runx2 is the “master regulator of osteogenesis”, as it is the main transcription factor that determines the commitment of mesenchymal progenitors towards the osteogenic lineage [11]. Runx2 promotes proliferation of mesenchymal cells, their commitment into osteoblast lineage cells and their differentiation into osteoblasts through the reciprocal regulation of hedgehog, fibroblast growth factor (*Fgf*), *WNT* and *PTHLH* signaling pathway genes and Dlx5. Runx2 enhances the proliferation of osteoblast precursors by modulating Fgfr2 and Fgfr3 [7].

Runx2 is responsible for the transactivation of major bone matrix protein genes via the osteoblast-specific cis-acting element (OSE) [6]. It forms heterodimers with the co-transcription factor core binding factor β (CBFβ) and regulates the expression of the osteoblast gene markers *COL1A1* (coding for collagen α-1(I) chain), *SPP1* (also known as *OPN*, coding for osteopontin), *BGLAP* (also named *OCN*, coding for osteocalcin), *IBSP* (also known as *BSP*, coding for integrin-binding sialoprotein), *OSX* and *ALPL* (also known as *TNAP*, coding for TNS-ALP, tissue-nonspecific alkaline phosphatase) [4,7,12,13,14,15,16,17].

Numerous transcription factors interact with Runx2 providing with costimulatory signals or repressing its function by affecting its DNA binding activity and/or transactivation potential [18]. Among these, Twist-related protein 1 and Twist-related protein 2 regulate Runx2 at the protein level by physically interacting with it and inhibiting its binding to DNA [19]. Also FoxO1 can interact with Runx2 and cooperates in the transcriptional regulation of osteoblastic markers [8]. Moreover, ATF4 is involved in indirect interactions with Runx2 during cell maturation to increase *OCN* expression, a marker of terminal osteoblast differentiation [20,21,22].

Runx2 is modulated by several post-translational modifications, such as acetylation, methylation, phosphorylation, SUMOylation, glycosylation, and ubiquitination, which affect the binding properties to genomic DNA [10,23,24]. Interestingly, the dynamic equilibrium among Runx2 acetylation, deacetylation, and ubiquitination is maintained by transmembrane and coiled-coil domains 1 (TMCO1)-mediated Ca^2+^ signaling [25]. Recent studies on Runx2 association with cis-regulatory elements (CREs) proved the association of Runx2 with the distal regulatory regions targeting skeletal genes and bone development and with the proximal regions (transcription start sites) of genes related to general cell activities, such as metabolic processes [24]. In line with this, Runx2 is associated with other genes that regulate the energy supply during osteogenesis [6]. Interestingly, Runx2 actively regulates the genes involved in glucose metabolism and energy homeostasis in mesenchymal cells [26]. Runx2 promotes glucose transporter 1 (*GLUT1*) gene expression, a glucose transporter that is responsible for the passive transport of glucose. Its uptake favors osteoblast differentiation and bone formation by inhibiting AMP-activated protein kinase (AMPK), therefore suppressing the AMPK-dependent proteasomal degradation of Runx2, and enhancing the activity of the mTORC1 pathway, and hence, causing protein synthesis, such as that of collagen [27]. Recently, a link between the odontogenic differentiation of human dental pulp stem cells (hDPSCs) and the GLUT1-mTORC1 axis has been discovered [28]. Interestingly, the reciprocal regulation of Runx2 and GLUT1 contributes to an amplification mechanism allowing proper bone formation [27].

Furthermore, a strong correlation between the RUNX family and the NFAT family has been found out in several cell types, as a Runx binding sequence has been discovered on the NFAT cytoplasmatic 1-3 (*NFATC1-3*) promoter, therefore directly regulating its transcription. The inhibition of the RUNX family determined the suppression of NFATC2 family at the transcriptional level and a reduction in the amount of the total NFATc family, determining a lower activation of downstream genes. Therefore, the RUNX–NFAT axis can be targeted to reduce cell growth and other functions [29].

### 1.2. Osterix Regulates Osteogenesis

Osx is localized to the nucleus and induces the expression of some mature osteoblast genes, such as *COL1A1*, *SPP1*, *BGLAP*, *IBSP*, *SPARC* (also known as secreted protein acidic and cysteine rich, *ON*, osteonectin), *IL10* (coding for interleukin-10), *MMP13* (coding for collagenase 3 or matrix metalloproteinase-13), *FMOD* (fibromodulin), *DKK1* (Dickkopf WNT signaling pathway inhibitor 1) and *ZBTB16* (Zinc finger and BTB domain containing 16). Osx can also interact with Runx2 to synergistically regulate gene transcription. *OSX* gene expression is regulated by two main pathways: one Runx2-dependent and one Runx2-independent [30]. Interestingly, Osx can form a complex with NFATc1, which promotes *COL1A1* gene expression [30,31,32].

*OSX* gene expression is regulated by miRNAs and long non-coding RNAs (lncRNAs) in an epigenetic manner, whereas the protein undergoes post-translational modifications which modulate the DNA binding and transcriptional activity of the transcription factor [30].

### 1.3. Other Factors Mediate Osteoblastogenesis and Osteogenesis

ATF4 regulates both the differentiation of osteoblasts and the activation of osteogenic genes by modulating *BGLAP*, *BSP*, and *OSX*. Interestingly, it is also involved in the amino acid import in osteoblasts and therefore in the regulation of protein diet and skeletogenesis [6].

The most important mediators of osteoblastogenesis and osteogenesis produced by immune cells are cytokines. Regulatory effects on osteoblastic genes are exerted by neutrophils, T cells, and macrophages, which mainly affect the BMPs and Wnt signaling [33,34].

Interestingly, calcium signaling influences both the cellular transcription activity and mineralization of ECM.

## 2. Cellular Calcium Signaling and Transcription Activity

**Figure 2:**
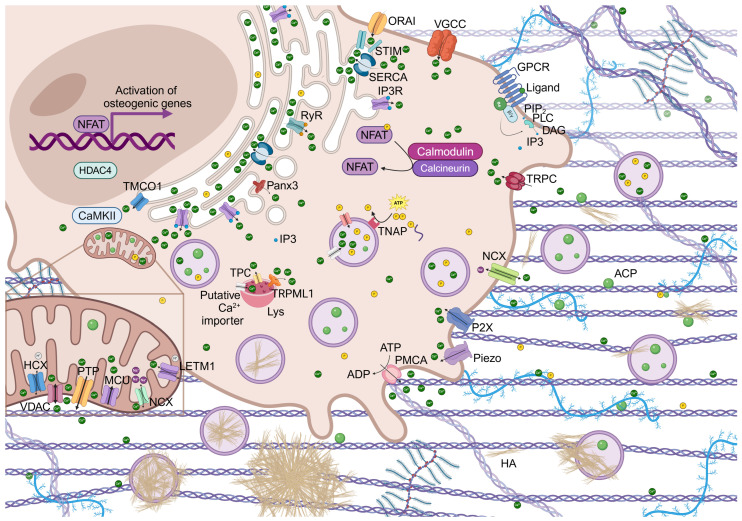
Schematic view of membrane channels, Ca^2+^ and inorganic phosphate (Pi) ion trafficking and amorphous calcium phosphate (ACP)/HA formation. Calcium transporters located at the PM, ER, mitochondria, and lysosomes (Lys) work coordinately to regulate specific functions, which include calcium sensing, calcium homeostasis, signal transduction, and organelle calcium loading. The Ca^2+^/CaN/NFAT signaling pathway promotes the process of osteoblast differentiation mediated by the nuclear translocation of NFAT. Ca^2+^ and Pi ions are represented as dark green and yellow spheres, respectively.

Oscillations of calcium concentration can activate many processes in cells through the Ca^2+^/calcineurin (CaN)/NFAT signaling pathway (Figure 2). It regulates growth and development of many types of cells, and, in particular, of osteoblasts and bone development and regeneration by influencing osteoblasts gene expression [2].

The intracellular calcium oscillations can be induced by important events such as the activation of a variety of signaling pathways, among which is the non-canonical Wnt signaling pathway. Interestingly, the activation of surface receptors determines the activation of phosphoinositide phospholipase C (PLC), which is responsible for the formation of inositol-1,4,5-trisphosphate (IP_3_). This binds to its receptor, IP_3_-receptor (IP_3_R), on the surface of the endoplasmic reticulum (ER), leading to the efflux of Ca^2+^ from it. Similarly, cADPR (cADP ribose), a cyclic derivative of ADP, can activate the ryanodine receptor (RyR) and promote Ca^2+^ efflux. The store calcium depletion determines the activation, oligomeration and translocation of the calcium sensor proteins, stromal interacting proteins (STIM1 or STIM2), on the ER to the ER-plasma membrane (PM) junctions, where they interact with the olfactory receptor class A related 1 (Orai1), determining the formation of the complex contained in the calcium-released activated channel (CRAC) and inducing a sustained influx of Ca^2+^. Calreticulin (CRT) is a Ca^2+^-binding multifunctional molecular chaperone in the ER, which takes part in the IP_3_-mediated Ca^2+^ release [35,36].

Moreover, the influx of Ca^2+^ is induced also by the environmental sensor calcium-sensing receptor (CaSR), which is a unique G protein-coupled receptor (GPCR), can sense the extracellular Ca^2+^ concentration, and can rapidly mobilize the intracellular Ca^2+^ flux. It is also induced by the voltage gated Ca^2+^ channel (VGCC), the ligand-gated Ca^2+^ channel, and the Na^+^/Ca^2+^ exchanger (NCX), which can regulate Ca^2+^ flowing into the cell [2,37].

Cytoplasmic Ca^2+^ can bind calmodulin (CaM), which undergoes a conformational change and can therefore bind to specific targets such as calmodulin kinase II (CaMKII) and CaN. CaN is activated and, in turn, promotes the dephosphorylation of NFAT transcription factors and their nuclear translocation in order to regulate gene expression [2].

Interestingly, it has been observed that a high extracellular concentration of 10 mM Ca^2+^ determined the nuclear translocation of NFATn [38,39]. Similarly, a concentration of 40 mM Ca^2+^ at the bone resorption site induced the increment of intracellular Ca^2+^ concentration [40]. The Ca^2+^/CaN/NFAT signaling pathway has been closely associated with the physiological activities of osteoblasts and compounds, which inhibit this signaling pathway and at the same time affect osteoblastogenesis [2].

In addition to CaN, CAMKII is also involved in the regulation of gene transcription. CAMKII is released from its autoinhibitory status, maximally activated and subjected to an intraholoenzyme autophosphorylation reaction. Phosphorylated CAMKII activates the CREB (cyclic AMP-responsive element-binding protein)/ATF and extracellular signal-regulated kinase (ERK) signaling pathways, which are involved in osteogenesis. The CaMKII/histone deacetylase 4 (HDAC4) pathway is mediated by TMCO1 and induces the enhancement of Runx2 stability and promotes bone formation. TMCO1 is an ER Ca^2+^ permeable channel that responds to ER Ca^2+^ overload, prevents intracellular Ca^2+^ stores from overfilling, and maintains calcium homeostasis in the ER through Ca^2+^ leakage from it [25].

### Role of NFAT Signaling in Bone Homeostasis

NFATs are a family of inducible transcriptional regulators identified in T cells over 35 years ago [41]. The *NFAT* gene family contains four classic members, *NFATc1* (also named *NFATc* and *NFAT2*), *NFATc2* (*NFATp*, *NFAT1*), *NFATc3* (*NFAT4*), and *NFATc4* (*NFAT3*), which are uniquely expressed only in vertebrates. The termination of the surface signaling and, in turn, a lower Ca^2+^ influx, leads to rephosphorylation of NFAT proteins by several kinases, among which are glycogen synthasekinase-3 (GSK3), cAMP-dependent protein kinase catalytic subunit alpha (PKA), mitogen-activated protein kinase kinase kinase (MEKK), and its export in the cytoplasm in an inactive state [37].

The CaN-NFAT signaling pathway plays a fundamental role in bone homeostasis. Osterix-, Runx2-, and Smad-dependent osteoblastic genes were upregulated by the overexpression of NFAT [31,37]. Interestingly, NFATc1 cooperates with osterix and Fos/Jun to activate target genes, such as *WNT4*, *Frizzled9* (Fz), *CCL8* (coding for C-C motif chemokine 8), and *OCN* [42]. Moreover, it has also been demonstrated that the induction of NFATc1 nuclear translocation may be associated with the increase in the expression of *WNT3A*, *WNT5A* and β-catenin [37,43]. Furthermore, it negatively regulates the expression of the Wnt inhibitory genes secreted frizzled-related protein 2 (*SFRP2*) and Dickkopf 2 (*DKK2*) [42].

Nevertheless, it has been proved that NFATc1 has a role in suppressing the promoter of osteocalcin during osteoblast differentiation, possibly mediated by HDAC3 interaction with Runx2 at the *OCN* promoter, and therefore repressing osteoblast maturation [37,44]. Indeed, it has been demonstrated that NFATc1 increased translocation and localization to the nucleus caused osteopetrosis, a rare disorder that causes bones to grow abnormally and with higher density and probability of bone breakage, by regulating chemokines and the Wnt pathways [42].

Furthermore, it has been found that NFATc2 may have a role in the regulation of bone homeostasis. In fact, Nell-1 is a growth factor that stimulates the expression of ECM proteins required for bone formation, is a target gene of Runx2, and can regulate NFATc2 [37,45,46,47,48]. Both *NFATc1* and *NFATc3* genes were upregulated when the extracellular calcium level was increased. NFATc3 may regulate bone homeostasis by leading to higher *RANKL* (also known as *TNFSF11*, coding for tumor necrosis factor ligand superfamily member 11) expression [38]. Activated osteoblastic RANKL promotes osteoblast differentiation through RANKL reverse signaling and the activation of *RUNX2* [49]. Moreover, the *NFATc3* expression is induced by the expression of *NFATc1* [38].

These data suggest that the involvement of CaN-NFAT pathway in the cellular transcription activity is finely regulated and different isoforms of calcineurin and NFAT, and different NFAT proteins exert distinct functions [37]. Therefore, calcium oscillations can influence gene expression and bone homeostasis.

Moreover, immunomodulation of MSCs by immune cells can promote osteogenesis by affecting NFAT. It has been demonstrated that T cells can promote proliferation and survival of bone marrow stromal cells (SCs) through the binding of CD40 ligand (CD40L), a surface molecule on T cells, to its receptor CD40 on SCs plasma membrane. CD40 signaling is activated and RANKL is produced [50]. CD40 lacks the kinase domain, and upon activation, recruits TNF-alpha Receptor Associated Factors (TRAFs) at the cytoplasmic domain. Both TRAFs-dependent and TRAFs-independent signaling activate phosphatidylinositol 3-phosphate (PI3K), Raf, Lyn, Syk, p38, Erk, and Protein kinase C (PKCs). CD40 can also activate NF-kB pathways, ATF2, ELK1, BLIMP-1, BATF, and NFAT, therefore modulating differential gene expression [51]. Interestingly, overexpression of NFATc1 and NFATc3 induced RANKL expression. Moreover, high extracellular Ca^2+^ determined activation of the CaN/NFAT pathway and the expression of RANKL [38,52,53].

Furthermore, NFAT signaling in osteoblasts induces the expression of chemokines, such as *CCL8*, that attract monocytic osteoclast precursors. Given the role of NFATc1 in osteoclastogenesis and in light of the above-mentioned studies, bone formation and resorption are integrated processes regulated by NFAT signaling [54].

## 3. Regulation of Intracellular Calcium Homeostasis

Ca^2+^ exerts a key role in the process of osteogenic differentiation. The concentration of cytoplasmic Ca^2+^ is normally around 0.1–0.2 µM. Calcium channels and transporters are necessary for calcium oscillations. Interestingly, mitochondria, lysosomes and ER are the organelles responsible for intracellular Ca^2+^ storage. There are crosstalks between organelles and plasma membrane Ca^2+^ channels in order to regulate cytosolic Ca^2+^ signals. For example, Ca^2+^ can modulate the proliferation and differentiation of osteoblasts through the mutual adjustment between CaSR, VGCC, and SOCE (store-operated calcium entry, a ubiquitous Ca^2+^ signaling pathway mediated by CRAC) [2,55]. Furthermore, Ca^2+^-permeable channels transient receptor potential (TRP), purinergic (P) (P2X and P2Y) receptors and Piezo channels on the PM can increase intracellular Ca^2+^ concentration. Moreover, activation of K^+^ channels inducing membrane hyperpolarization indirectly promotes Ca^2+^ signaling in osteoblasts linage cells [36].

Furthermore, IP_3_R, RyR, TMCO1 and pannexin3 (Panx3) Ca^2+^ channel in the ER transmembrane, Na^+^/Ca^2+^ exchanger (NCX), Na^+^-Li^+^/Ca^2+^ exchanger (NCLX), Na^+^-independent Ca^2+^ exchanger (H^+^/Ca^2+^ exchanger, HCX) and the Permeability Transition Pore (PTP) in mitochondria and the transient receptor potential mucolipin subfamily 1 (TRPML1 or ML1) and two-pore channels (TPCs) in lysosomes can favor the increment of Ca^2+^ in the cytosol [25,36,56,57,58,59,60,61].

The extracellular Ca^2+^ concentration is normally around 1–2 mM. Its concentration is 0.5–1 mM in the ER, 0.1 µM in the mitochondria and 2 µM in the lysosomes [62]. It has been demonstrated that extracellular Ca^2+^ concentration of 1.8–7.8 mM induced the increase in cell size and proliferation. Higher extracellular values of Ca^2+^ concentration of 10–15 mM Ca^2+^ determined the activation of downstream MAPK signaling pathways mediated by this ion, which promoted the expression of osteogenic differentiation related genes. Nevertheless, Ca^2+^ concentration of 50 mM hindered the normal adhesion of cells [2].

The increase in calcium flow can generate subplasmalemmal high Ca^2+^ microdomains (HCMDs). Depending on the cell type and relative position of organelles, channels and other molecules, physiological responses, such as exocytosis, contraction or cell growth, can be induced or Ca^2+^ can be taken up by mitochondria through the mitochondrial Ca^2+^ uniporter (MCU) or by ER in order to avoid progression of the Ca^2+^ wave towards the cell core [63]. Mitochondrial Ca^2+^ accumulation is usually transient as this organelle is normally empty but it can accumulate large amounts of Ca^2+^ when its concentration is above 1 µM, nevertheless its overload (over 3 mM/mg protein) can determine damages to the organelle and can trigger, in turn, apoptotic mechanisms [63,64,65]. In addition to MCU, the leucine zipper, EF-hand containing transmembrane protein 1 (LETM1) allows the transport of calcium ions inside the mitochondria and of protons outside the mitochondrial matrix [66]. The voltage-dependent anion channel (VDAC) is located on the outer membrane of the mitochondria and is responsible for Ca^2+^ ions and ATP transport [67].

**Figure 3:**
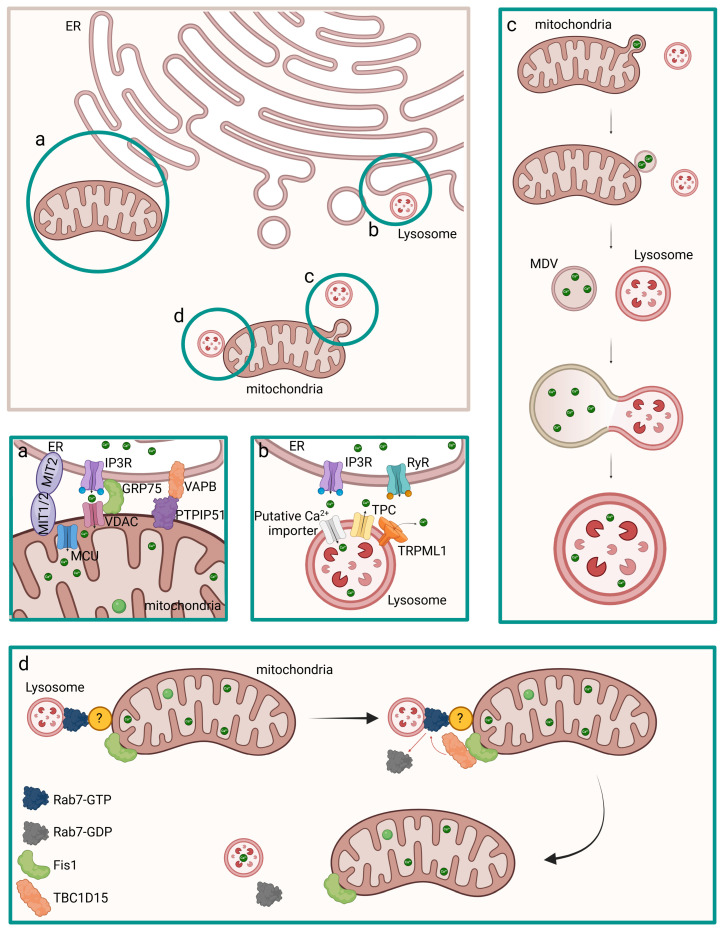
Coordination of Ca^2+^ homeostasis in ER and other intracellular organelles, such as mitochondria and lysosomes. Ca^2+^ ions transfer can occur from ER, which can interact both with mitochondria (**a**) and lysosomes (**b**), or from mitochondria to lysososomes/vesicles through direct interaction (**d**) or MDVs (**c**) (brown square). The regulation of Ca^2+^ homeostasis in the above-mentioned organelles is shown separately in the relative green squares labeled with a, b, c, and d. (**a**) Ca^2+^ is released from the ER through IP_3_R and it is internalized by mitochondria through VDAC on the outer membrane and MCU on the internal mitochondrial membrane. The interaction between the IP3R and VDAC is mediated by GRP75. The interaction between the two organelles is mediated by MFN2 on the ER and MFN1 or MFN2 on the mitochondria and by VAP8 on the ER and PTPIP51 on the mitochondria. (**b**) Ca^2+^ is released from the ER through IP_3_R, and it is internalized by the lysosome through Ca^2+^ importers. (**c**) Mitochondria releases MDVs, small vesicles that shuttle mitochondrial constituents to other organelles, which contain Ca^2+^ ions. MDVs deliver Ca^2+^ ions to lysosomes. (**d**) Inter-organelle transfer of Ca^2+^ from mitochondria to lysosomes is mediated by Rab7. Untethering of the two organelles occurs due to Fis1 located on the mitochondria which recruits TBC1D15, a Rab7 GAP. The dark yellow sphere labelled with “?” represents an unknown protein.

An important crosstalk between mitochondria and ER is due to dynamin-related mitofusins, which are part of the tethering mechanism between these two organelles, and determines the release of Ca^2+^ from the ER through IP_3_Rs coupled to the mitochondrial uptake through MCU and the mitochondrial voltage-dependent anion channel 1 (VDAC1) on the internal and outside mitochondrial membranes, respectively (Figure 3) [63]. The MAM (mitochondrial-associated endoplasmic reticulum membranes) regions contain the ER proteins mitofusin 2 (MFN2) and vesicle-associated membrane protein (VAMP)-associated protein B (VAPB), which interact with the mitochondrial mitofusin 1 (MFN1) or MFN2 protein and the protein tyrosine phosphatase-interacting protein 51 (PTPIP51) [68,69,70]. The cytosolic glucose-regulated protein 75 (GRP75) is part of the physical tethering of ER and mitochondria and mediates the interaction between VDAC and IP_3_R [71].

Intracellular Ca^2+^ is also decreased by NCX at the PM and by Ca^2+^ extruding ATP-driven enzymatic pumps such as PMCA at the PM, SERCA at ER, and SPCA at the Golgi [72]. It is likely that a mechanism to transport Ca^2+^ from the cytosol inside lysosomes such as Ca^2+^ exchangers or Ca^2+^ ATPases might exist and that a lysosomal-ER crosstalk might be a most critical Ca^2+^ source [73,74].

Interestingly, the ER drives calcium refilling of lysosomes upon contact with this organelle. In contrast with what was previously suggested, lysosomal acidification mediated by the V-ATPase H^+^ pump was not associated with Ca^2+^ refilling, whereas pharmacological depletion or chelation of ER Ca^2+^ or antagonists of ER IP_3_Rs were able to prevent Ca^2+^ refilling of lysosomes. In addition to Ca^2+^ uptake through endocytosis, most of lysosomal Ca^2+^ requires the activity of lysosomal Ca^2+^ channels and it has been hypothesized that a functional interaction between ER and lysosomes could be the direct source of Ca^2+^ to lysosomes through the formation of nanojunctions composed of IP_3_R on the ER, which is responsible for producing a local high Ca^2+^ concentration, an unknown low-affinity Ca^2+^ transport mechanism on the lysosome, and unidentified tether proteins, which link the ER and lysosomal membrane proteins [73,75].

It has been recently demonstrated that additional mechanisms of mitochondrial disposal and mitochondrial quality control (MQC) recycle whole damaged mitochondria or mitochondrial fragments through the production of mitochondria-derived vesicles (MDVs). These organelles are generated directly through the budding from the mitochondrial membranes to extrude damaged content and can fuse to vesicles deriving from the endosomal–lysosomal system, such as multivesicular bodies (MVBs) and lysosomes. It has been hypothesized that MDVs can contribute to regulate calcium homeostasis by containing Ca^2+^ ions and fusing with lysosomes [76,77,78,79]. Moreover, direct interaction between mitochondria and lysosomes via non-degradative process has been identified and may favor the inter-organelle transfer of calcium. Interestingly, lysosomal guanosine triphosphate (GTP)-bound Rab7 promotes contact formation and tethering to the mitochondria, which could be mediated by Rab7 effector proteins. The recruitment of TBC1D15, a Rab7 GTPase-Activating Protein (GAP), to mitochondria mediated by the outer mitochondrial membrane protein Fis1 leads to untethering [80].

The cytosolic calcium ion concentration is controlled by the activation of membrane pumps and channels, the calcium ion capture by subcellular organelles, and the action of calcium-binding proteins in the cytosol. Annexins are a group of calcium-binding proteins that have an important role in the mineralization process by working as nucleating centers for extracellular mineral deposition and by providing critical sites for intracellular calcium ion trafficking and cluster formation. Annexins are localized on intracellular organelles, the PM and MVs [81]. Proper regulation of intracellular concentration of Ca^2+^ or extracellular Ca^2+^ can enhance osteogenic differentiation and mineralization [2]. The involvement of mitochondria and ER is important in regulating Ca^2+^ transport and accumulation and the initiation of the mineralization process [82].

## 4. Formation of the Prebone and Bone Matrix

### 4.1. Osteoid

Osteoid, which is an unmineralized precursor of bone, is secreted at the sites of new bone formation and is normally surrounded and controlled by an organized epithelioid structure made of osteoblasts. The prebone (osteoid) ECM is an organic matrix made of structural proteins (collagens), which compose dense layers that alternate parallel and orthogonal to the axis of stress loading, non-collagenous matrix proteins (osteonectin, osteocalcin, osteopontin, sialoprotein), proteoglycans (PG), such as decorin, lumican, biglycan and others, and glycosaminoglycans such as chondroitin sulfate and hyaluronan, which are macromolecules that provide the framework for collagen formation. The collagen–proteoglycan matrix is able to bind calcium salts. Some of the non-collagenous proteins play important regulatory and structural roles in order to regulate bone formation and regeneration [54,83,84].

### 4.2. Bone Formation and Mineralization

**Figure 4:**
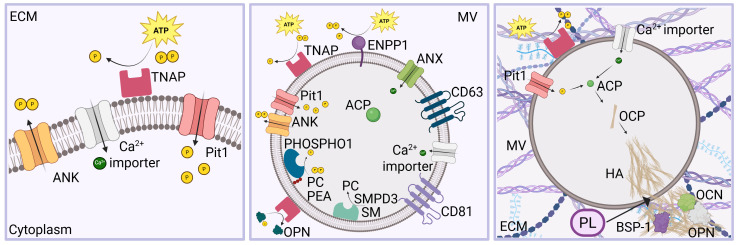
The mineralization process is regulated by enzymes and transporters. Pi is produced by TNAP on the PM, while channels are responsible for Ca^2+^ and Pi intracellular homeostasis (**left**). MVs are characterized by the presence of CD9, CD63, and CD81 tetraspannins on the membrane. SMPD3 (sphingomyelin phosphodiesterase 3) catalyzes the hydrolysis of sphingomyelin (SM) to form ceramide and phosphocholine (PC), whereas TNAP, ENPP1, and PHOSPHO1 are responsible for the formation of Pi. PHOSPHO1 is a phosphatase with high activity toward phosphoethanolamine (PEA), PC, and pyrophosphate (PP). ANX, Pit1, and Ca^2+^ importers regulate the transport of ions inside the MVs (**center**). The mineralization process takes place in MVs, which are released by osteoblasts. Ca^2+^ and P_i_ are internalized and form ACP, which is transformed into OCP and subsequently into HA. This penetrates through the vesicle membrane thanks to phospholipases, leading to the breakdown of the MV membrane. The following nucleation of HA crystals and spreading into the ECM is regulated by extracellular Ca^2+^, P_i_, H^+^, BSP-1, OCN, and OPN (**right**).

Osteoid undergoes mineralization to form mature bone. This process is promoted by vesicles of 30 to several hundreds of nanometers in diameter named MVs which can be classified as extracellular vesicles (EVs) produced during specific cellular processes in line with the current definition of EV by the International Society for Extracellular Vesicles (ISEV) [85]. MVs are particles released from osteoblasts during osteogenesis, are delimited by a lipid bilayer, cannot replicate on their own, and contain molecules, such as annexin, TNAP, and CaP (Figure 4). It has been hypothesized that both MVs and EVs could present similar biogenesis pathway, size, and morphology, but they exhibit different roles [86].

The calcification process starts with the formation of amorphous calcium phosphate (ACP) due to an aggregation process of Ca^2+^ and inorganic phosphate (Pi) ions, which transform to more stable crystalline phases, such as octacalcium phosphate (OCP, Ca_8_(HPO_4_)_2_(PO_4_)_4_·5H_2_O), calcium-deficient apatite (CDHA, Ca_9_(HPO_4_)(PO_4_)_5_(OH)), and hydroxyapatite (HA, Ca_10_(PO_4_)_6_(OH)_2_). HA is the major component of vertebral tooth and bone tissue. As the matrix matures, HA microcrystals are organized into more complex structures [83,87,88].

The crystallization process occurs within vesicles, followed by the formation of calcified nodules [83,87,88]. In fact, after the release of these vesicles, needle-like crystals pierce the membrane of MVs, and further accumulation of ACP and HA crystals in the extracellular matrix is induced due to the presence of free ion complexes [89]. Both the HA crystals and the free ion complexes serve as foci for mineral formation [2,7,36,55,72]. A further step of mineralization, which determines the formation of the bone (mineralized) ECM, is the formation of mineralized nodules and calcifying globules with deposition within intrafibrillar and interfibrillar spaces of collagen. Glycoproteins, growth factors (GFs), and other non-collagenous proteins can influence mineralization [89]. Calcification of the matrix occurs experimentally only at mildly alkaline levels of pH [83,88]. The formation of prebone and bone matrix occur at the apical surface of osteoblasts [83].

### 4.3. Regulation of Phosphate Ions Homeostasis

Cellular influx of Pi ions occurs through multimeric membrane exchanger/transporter proteins, including family 20 member A1/sodium-dependent phosphate transporter 1 (SLC20A1/PiT1), family 20 member A2/sodium-phosphate transporter 2 (SLC20A2/PiT2), family 34 member A1/sodium-phosphate co-transporter IIa (SLC34A1/NaPi-IIa) and family 34 member A2/sodium-phosphate co-transporter IIb (SLC34A2/NaPi-IIb) [83]. Efflux of Pi from the cells occurs through the XPR1 transporter protein. Ionic homeostasis of the cytosol is regulated also by other types of exchanger proteins, such as sodium/hydrogen and sodium/potassium membrane pumps [83,90,91,92,93]. Pi are present intracellularly at a higher concentration compared to the plasma [94]. Pi can be localized in the cytoplasm, in the ER and in mitochondria [95,96].

TNAP, phosphoethanolamine/phosphocholine phosphatase 1 (PHOSPHO1) and Ectonucleotide pyrophosphatase/phosphodiesterase I (ENPP1) are responsible for the Pi production. Interestingly, ENPP1 is a membrane-bound glycoprotein that can catalyze ATP and generate PPi and is coupled with TNAP, a glycosylphosphatidylinositol anchor enzyme, which can hydrolyze PPi into Pi to promote mineralization. TNAP is localized in large amounts on the PM and on the vesicles membrane; ClC3 and ClC5 can alter its normal activity [83,89]. Differently, PHOSPHO1 can hydrolyze PPi into Pi inside MVs. Moreover, ANK, which is encoded by the progressive ankylosis gene (Ank), is a non-enzymatic PPi channel that can enable PPi exit from MVs, whereas Pi can be internalized into MVs by sodium-inorganic Pi co-transporters Pit1 and Pit2 [89]. Moreover, TNAP might be able of recruiting calcium ions. It was also found in and around the calcified nodules. The Ca^2+^ and Pi ions were localized differently, at proteoglycan sites and collagen fibril structures, respectively, in the uncalcified matrix, therefore limiting the CaP production while Ca^2+^/Pi colocalization was observed in and around the calcified nodules. The activity of TNAP is fundamental for the ECM mineralization [89,97].

### 4.4. Mechanisms Driving Mineralization and Resorption

The process of mineral deposition produces acid and requires removal of protons for driving mineral precipitation and formation of an extremely dense mineral. This occurs thanks to Cl^−^/H^+^ exchangers that move acid into osteoblasts, whereas acid extrusion at the basolateral membranes is mediated by sodium–hydrogen exchangers 1 and 6. This process and the osteoblast Pi transport are regulated by the sodium–hydrogen exchanger regulatory factor-1. The process of mineral deposition might also require K^+^ channels, which might correlate with ClC exchangers and other ion transporters, such as an outward pump of net sodium at the basolateral surface and the reuptake of chloride at the apical surface. In addition to acid uptake from bone matrix, water is removed from the dense collagen hydrogel forming the osteoid. Tissue arrays suggest that osteoblasts express aquaporins [83].

Due to matrix growth, osteoblasts undergo either apoptosis or terminal differentiation to form osteocytes which are incorporated into the matrix and communicate with each other and with the cells surrounding the bone matrix through cell processes within canaliculi in the matrix [83,98].

The alkaline nature of bone formation is counteracted by the essential acidic resorptive microenvironment of bone degradation, where the addition of protons is necessary for the recovery of ionic phosphate and calcium during bone resorption. This process occurs in confined areas (resorption lacunae) surrounded by osteoclasts, which resorb bone via the ruffled border, a distinct bone-facing membrane domain where lysosomes fuse and promote acidification and the release of lysosomal proteases [99].

The balance between bone mineralization (alkaline environment) and resorption (acidic environment) is modulated by osteoblasts and osteoclasts, respectively [83].

## 5. Biogenesis, Trafficking and Release of MVs

In addition to limited amounts of calcium ions, which diffuse through the membrane, and specific transport systems, which regulate the cation passage into the cell, specific cellular pathways are responsible for Ca^2+^ accumulation, trafficking and discharge from intracellular organelles, such as vesicles. Mineralization occurs through mineral delivery and deposition as packages of ACP nanospheres, which transform into structures of crystalline apatite (HA) within the collagen matrix [100].

**Figure 5:**
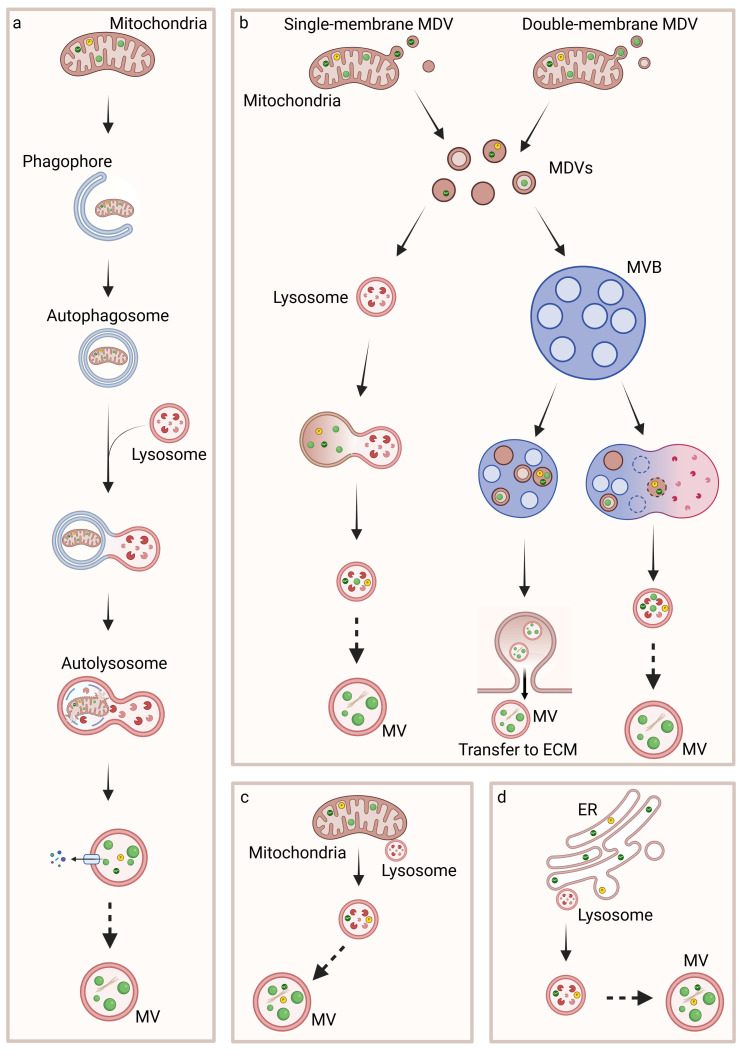
Biogenesis of MVs and intracellular biomineralization process. Ca^2+^, Pi, and ACP are produced in mitochondria and transferred to intracellular MVs through mitophagy (**a**), MDVs (**b**) or interaction with lysosomes (**c**). MVs are also generated through the transfer of Ca^2+^ and Pi ions from ER to lysosomes (**d**). It remains unclear whether autolysosomes and lysosomes undergo further steps in order to acquire the typical characteristics of MVs.

Several findings on the physiological process of biomineralization have been recently published; however, a unique process of MVs biogenesis, CaP formation, and secretion of vesicles has not been associated with it. Indeed, it has been demonstrated that MVs could form from vesicles, likely lysosomes, that come into contact with mitochondria in order to internalize Ca^2+^ before being released, from lysosomes that fuse with autophagosomes containing mitochondria which present ACP in the mitochondrial matrix, from multivesicular bodies (MVBs) and from vesicles at later steps of intracellular trafficking [64,77,78,79,80,82,89] (Figure 5).

**Figure 6:**
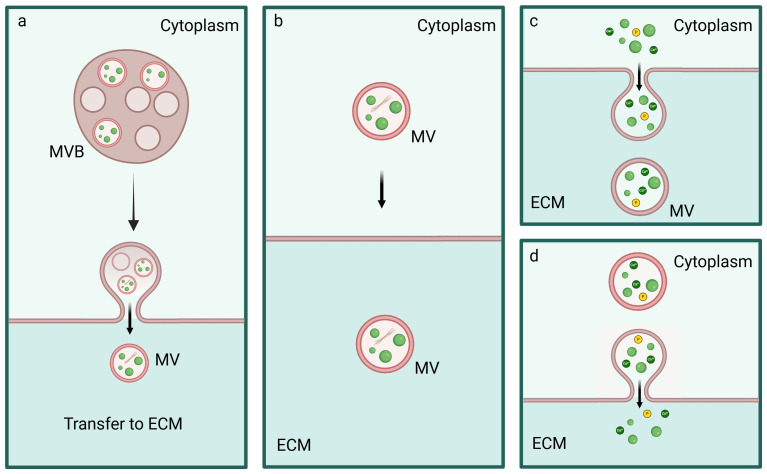
Release of Ca^2+^ and Pi ions, ACP, and MVs. MVBs (**a**) or MVs (**b**) interact with the PM and lead to the release of Ca^2+^ and Pi ions, ACP, and HA in the ECM. Moreover, free Ca^2+^ and Pi ions and ACP can be released in the ECM as microsomes (**c**) or through exocytosis (**d**).

In line with these works, ACP/HA could be released in MVs secreted from MVBs similarly to exosomes or through ACP/HA-containing MVs that interacts with the PM and are secreted in the ECM [101,102]. Further release of Ca^2+^, Pi ions, and ACP can occur through exocytosis or through polarized budding and pinching-off processes with the release of MVs, similarly to ectosomes (Figure 6) [77,78,103,104,105,106].

### 5.1. Inter-Organelle Communication and Physicochemical Structure Development of CaP

The process of formation of ACP precursors supports the view of intracellular initiation of biomineralization in organelles followed by the release of vesicles in the ECM.

It has been observed that both ER and mitochondria contain both Ca^2+^ and Pi ions [95,96,104]. Interestingly, it has been hypothesized that the initial process of formation of minerals occurs in the ER and a first contact between ER and mitochondria is required for the formation and accumulation of CaP in mitochondria. At mitochondria-ER contact sites, (MERCs) Ca^2+^ is accumulated at a tenfold higher concentration than normal in the bulk cytosol microdomains, which are involved in the ions and molecular exchange between the two organelles leading to the ER promoting the entry of Ca^2+^ into mitochondria. Pi can be transported independently through MERCs. In particular, the transport of Pi could occur through the glucose-6-phosphatase (G6Pase) transport system in the ER, which hydrolyzes glucose-6-phosphate to glucose and Pi. Pi can, in turn, be transported through the transport system termed T2 on the ER to the cytoplasm and pass effortlessly through the outer mitochondrial membrane (OMM) due to its high permeability. Subsequently, the phosphate carrier (PIC) on the inner mitochondrial membrane (IMM) barrier is responsible for the uptake of Pi. Due to the transport of Ca^2+^ and Pi from ER to mitochondria, ACP can be formed in the mitochondrial matrix and in the cytoplasm after the first part of the transport [89].

Furthermore, a second inter-organelle communication is necessary between mitochondria and vesicles in order to have CaP accumulated in vesicles and the nucleation and maturation of minerals. In the 1960s, insoluble CaP electron-dense granules reported as ACP precursors were detected in mitochondria [107]. In the same period, it was discovered that the earliest mineral deposits were in contact with membrane-bounded TNAP-positive vesicles initiated from intracellular structures [89,108,109]. The transport of Ca^2+^ and Pi ions or ACP from mitochondria to vesicles has been observed and could occur through mitophagy, direct contact between mitochondria and lysosomes or other vesicles, and the formation of MDVs and subsequent contact with other vesicles [64,77,78,79].

Mitophagy is a cytoprotective mechanism and a type of macroautophagy that removes dysfunctional or superfluous mitochondria by transporting them to lysosomes for degradation [110]. The first step of this process is the engulfment of mitochondria in a cup-shaped sequestering compartment named phagophore, which matures into the double-membraned vesicle named autophagosome. ACP precursors localized in the mitochondrial matrix are therefore transported to autophagosomes, which are conveyed to lysosomes. The outer membrane of the autophagosome fuses with the lysosome membrane to form an autolysosome, an acidic degradative hybrid organelle, while the inner membrane and the content of the autolysosome are degraded by the lysosomal enzymes and recycled. In these organelles, ACP precursors coalescence to form larger intra-vesicular granules [64].

The transport of Ca^2+^ and Pi ions or ACP from mitochondria to vesicles may also occur through MDVs, which are a set of intracellular vesicles of 70–150 nm in diameter of mitochondrial origin. They are constitutively produced under basal conditions and can be highly produced under specific conditions. MDVs shuttle mitochondrial cargoes to peroxisomes, lysosomes, and MVBs and are single- or double-membrane organelles, budding from the OMM or shedding from both the OMM and IMM, respectively. The double-membrane MDVs contain portions of mitochondrial matrix and could contain ACP. Both subtypes of MDVs are directed to lysosomes for degradation or lysosomal exocytosis or to MVBs for the exocytosis of MVs [76,111].

Scanning electron-assisted dieletric microscopy and super-resolution microscopy have been used to evaluate the intracellular MVs transport and secretion at a nanolevel resolution. Interestingly, lysotracker-positive vesicles were able to fuse with calcein-positive vesicles adjacent to mitochondria and the newly formed vesicles moved towards the extracellular space. Calcein is a membrane-impermeable calcium binding fluorescent probe, and mitochondria have been proposed to be the source of these static calcium-containing vacuoles adjacent to them. The fusion of these vesicles with lysosomes, characterized by the cationic dye lysotracker that preferentially accumulates in acidic lysosome and by the lysosomal resident marker protein 1 (LAMP1) might determine the formation of MVs containing ACP, which are directed to the periphery in order to be secreted. When the osteoblasts were treated with a lysosomal proton adenosine triphosphatase inhibitor, bafilomycin A1 (BafA), lysosomal acidification was completely abolished and the formation of the hypothesized MVs was also negatively affected. Nevertheless, when the cells were treated with an exocytosis inhibitor, vacuolin-1 (Vac-1) or with the inhibitor of lysosomal exocytosis endosidin 2 (ES2), which targets the exocyst complex subunit, intracellular accumulation of vesicles and calcein occurred and mineralization was blocked [78].

Ca^2+^- or ACP-containing lysosomes or lysosome-related organelle may fuse with other vesicles, such as the TNAP-positive vesicles originating in the secretion pathway, in order to acquire the typical markers and the specific molecules of MVs. Furthermore, acidocalcidomes, acidic calcium stores, which are another form of the lysosome, have been identified in human cells in addition to bacteria and can take part to the process of MVs biogenesis. These organelles are rich in phosphorus compounds and their acidity is maintained by proton pumps [112].

Interestingly, the interaction between ER and lysosomes or other vesicles can promote the formation of MVs thanks to the transport of Ca^2+^ from the ER through IP_3_R and its internalization by the vesicles through Ca^2+^ importers [73,75].

Furthermore, it has been hypothesized that MVs can originate from autophagosomes that fuse with endosomes leading to the formation of amphisomes or from other vesicles, besides MDVs, that can provide MVBs with Ca^2+^ and Pi ions and ACP [113,114,115]. In fact, ion loading by endosomes could occur thanks to specific proteins, such as nucleobindins, calbindins, and calreticulins located in the cytoplasm which interact with Ca^2+^ and favor its transit to endosomes [116,117,118]. Moreover, endosomes could accumulate ions from other sources, such as from ER, mitochondria, or from the cytoplasm through channels [114].

Another mechanism through which ACP precursors are transported from mitochondria through vesicles to the ECM is overloaded Ca^2+^ influx into mitochondria, which triggers the opening of mPTP, leading to the release of pro-apoptotic molecules and activation of downstream CASPs, inducing ROS generation and apoptosis. Indeed, mitochondrial disfunction leads to apoptotic cell death, the formation of apoptotic bodies, which are heterogeneous vesicles containing ACP/HA, and promotion of calcification [101,102].

In light of what has previously been described, it has been hypothesized that MVs have a mixed origin, and an overlap of pathways can occur. Moreover, in addition to the MVs associated with activities of the cellular endosomal-autophagic system [64,78,89,113,115], several works report MVs as derived from protrusions, blebs, or buds from the PM [109,119,120,121,122].

### 5.2. Regulation of MVs Biogenesis and Intracellular Trafficking

Ras-associated binding (Rab) proteins are guanine nucleotide-binding proteins which regulate intracellular membrane trafficking by orchestrating the biogenesis, transport, tethering, and fusion of vesicles with a target membrane. Rabs cycle between two states, an active membrane-bound (GTP-loaded) form and an inactive unbound (GDP-loaded) form. Their cycling is regulated by GTPase-activating proteins (GAPs) for GTP hydrolysis and GTP exchange factor proteins (GEFs) for nucleotide exchange. More than 60 human Rab proteins have been identified. They decorate the surface of specific organelles and recruit effector proteins in order to modulate the membrane trafficking [123].

Other molecules are involved in the regulation of the intracellular trafficking, such as SNARE (Soluble NSF Attachment Protein REceptor) proteins which are fundamental for membrane fusion [124].

It has been demonstrated that Rab4, Rab5, Rab11, Rab14, Rab18, and Rab21 were present in matrix vesicles isolated from SAOS osteoblast-like cells [82,125].

Interestingly, it has been proved that Rab11 is a fundamental molecule for regulating the trafficking of sortilin, a key regulator of smooth muscle cell (SMC) calcification via its recruitment to MVs. Sortilin mediates the loading of TNAP from the Golgi apparatus to MVs. TNAP is able to induce high mineralization competence in the ECM, therefore determining the calcification [126]. Rab11 is also involved in the regulation of the fusion of lysosomes and late endosomes (LE) with Rab11-positive vesicles in order to promote lysosomal exocytosis. Interestingly, both Rab11a and Rab11b have been identified as regulators of Ca^2+^-induced lysosome exocytosis. The transient interaction occurs at the cell periphery and it is mediated by the exocyst subunit Sec15, a Rab11 effector. It has been hypothesized that Sec15 might function independently of the exocyst complex. Therefore, it could have an important role in mediating the transport of Rab11-positive vesicles towards the periphery and late endocytic compartments along the actin cytoskeleton by interacting with myosin V. Afterwards, Rab11 binds Rab3a and GRAB, a Rab3a GEF. In particular, Rab3a GEF activates Rab3a which, in turn, binds to the LE and lysosomes and recruits the non-muscle myosin heavy chain IIA (NMIIA, encoded by *MYH9*) and synaptotagmin-like protein 4a (Slp-4a, encoded by *Sytl4*) in order to determine the positioning and exocytosis of lysosomes. It has been demonstrated that this process is triggered by Ca^2+^ as treatment with the Ca^2+^ ionophore ionomycin impairs lysosome exocytosis by hindering the interaction between Rab11-positive vesicles with lysosomes and LE [127].

Furthermore, it has been demonstrated that the active form of Rab7 promotes contact formation and tethering between Rab7-positive lysosomes and mitochondria in order to promote inter-organelle transfer of metabolites, including lipids, calcium, or iron, whereas the mitochondrial TBC1D15, a Rab7 GAP, determines Rab7 inactivation and contact untethering [80].

Interestingly, Rab32 and phosphofurin acidic cluster protein 2 (PACS2) can influence the localization of calnexin (CNX) and, therefore, the transport of Ca^2+^ from ER to mitochondria. This occurs through the opening of the IP_3_Rs, which is regulated by a set of proteins present at or recruited to MAMs, including CNX, the Sigma non-opioid intracellular receptor 1 (Sigma1R), presenilin 1 and 2 (PS1 and PS2) [128].

Further studies are necessary to identify other Rab proteins and relatives SNAREs involved in the biogenesis, ion loading and trafficking of MVs.

## 6. Discussion

The initiation of osteogenesis is related to the differentiation of mesenchymal stem cells into osteoblasts. This process is based on a gene expression program associated with the bone matrix production. Interestingly, oscillations of intracellular Ca^2+^ can influence the expression level of specific genes, such as RUNX2 and OSX [31,37,83].

Osteoblasts are cuboidal cells that display large amounts of rough ER and mitochondria and secrete bone organic matrix [129]. Bone formation is characterized by the release of MVs at the apical surface of osteoblasts and mineralization is driven by both active and passive transport and pH control [83]. Therefore, both osteoblast differentiation and bone matrix synthesis are coupled with the regulation of calcium flow. While there are several studies proving the influence of Ca^2+^ oscillations on gene expression and osteblastogenesis, several aspects of the biomineralization process are not yet clarified.

Many studies reported that CaP within intracellular MVs was mainly amorphous; nevertheless, it has also been found that extracellular MVs contained crystallized CaP, suggesting that CaP forms the more stable form HA in secreted MVs whose membrane is disrupted by the mineralization process determining the release of HA in the ECM [79].

Calcium phosphate can crystallize to form HA in neutral to basic pH. It has been demonstrated that OCP forms preferentially at pH 5.5 or higher, whereas HA is formed at pH above 7 (preferentially around pH 7.4–8) [130]. Interestingly, the pH values of the eukaryotic compartments and organelles have been studied and intracellular cytosolic pH is approximately 7.2. This value varies significantly between the different organelles. The pH is generally 6.3 in early endosomes, 5.5 in MVBs and late endosomes, and 4.7 in lysosomes. Like endocytic organelles, recycling endosomes have a pH of 6.5, while secretory granules have a pH of 5.5. In contrast the pH value is 7.2 in the ER and 8 in mitochondria. pH values are maintained by a balance between ion pumps, leaks, and internal ionic balance. Interestingly, endosomal pH can be modulated by affecting the membrane potential. Compared to the cytosolic pH, the pH of ECM is slightly alkaline with a value of 7.3–7.4 [131].

ACP has been detected in cytosol, mitochondria, and vesicles, including MVs [79,114,132]. ACP might also be formed in intraluminal vesicles (ILVs, vesicles located inside MVBs); however, it has not yet been clarified how MVs acquire a higher pH and other specific markers when they originate from acidic organelles such as lysosomes, lysosome-related organelles, and acidocalcisomes and whether TNAP is necessary for promoting ACP formation and accumulation. It can be hypothesized that acidic vesicles could interact with other compartments, such as more alkaline compartments, MDVs, and TNAP-positive secretory granules. In support of this, Rab11 could mediate the transport of TNAP from the secretory pathway to Rab3a-positive LE, lysosomes, or lysosomes-related vesicles before exocytosis [126,127]. The acquisition of TNAP on MVs might occur also during the secretion step by transferring TNAP from the PM to the released vesicle.

In support of the relationship between autophagy, and relative lysosome-related vesicles that are produced, and bone mineralization, it has been proved that there is a marked increase in autophagy during osteoblast differentiation. In fact, it has been demonstrated that there is an association between genes involved with the regulation of the autophagic pathway and bone mineral density and a change in the lipidated form of LC3-II protein at the onset of mineralization [133,134]. Furthermore, autophagosomes containing Ca^2+^ and Pi determined the formation of calcified MVs and promoted the extracellular mineralization [135].

Another interesting hypothesis is that MDVs could constitute the main source of MVs. Nevertheless, specific membrane composition and cargo selection have been reported for single- and double-membrane MDVs and further studies may clarify the correlation between these two subsets of MDVs and MVs biogenesis, the trafficking and further interaction of MDVs with other organelles prior to the unloading into the ECM [111].

It has been demonstrated that the Ca^2+^ load in lysosomes is not pH-dependent, while MVs formation is affected by lysosomal pH [73,75,78]. In light of these findings, it can be hypothesized that H^+^ flux is important for ion homeostasis in lysosomes.

Similarly to the vesicles in the endolysosomal-autophagic system, regulatory proteins such as Rabs and their effectors could regulate membrane fusion between MVs and other compartments, the expression of pumps and channels, and the presence of specific enzymes in MVs.

The process of mineral formation begins in the cell and terminates in the ECM. It is a multistep process that includes biogenesis, ion loading, trafficking, and the release of MVs. This process is highly regulated and involves several molecules responsible for the maintenance of intracellular ions homeostasis, vesicle trafficking, and pH control, those modulating the actin fiber distribution in correlation with the fluidity of the cell membrane, and many other cell components [136]. Nevertheless, oscillations of calcium flow have been highlighted as a key player in the mechanism of biomineralization, as Ca^2+^ signaling mediates gene expression and Ca^2+^ transport affects CaP formation, MV biogenesis and release, and the deposition and mineralization of collagen fibers.

## Data Availability

No new data were created or analyzed in this study.
